# Acute Kidney Injury in the Emergency Department: Key Predictors for Early Renal Replacement Therapy

**DOI:** 10.5152/eurasianjmed.2026.251272

**Published:** 2026-03-02

**Authors:** Ali İlker Akdoğanlar, Salim Satar, Selen Acehan, Müge Gülen, Engin Onan, Sedat Kuleci, Sarper Sevdimbaş, Cumali Kuş

**Affiliations:** 1 Emergency Medicine Clinic, Karşıyaka State Hospital, Adana, Türkiye; 2 Health Sciences University, Adana City Training and Research Hospital, Emergency Medicine Clinic, Adana, Türkiye; 3 Department of Nephrology, Başkent University Adana Dr. Turgut Noyan Training and Research Hospital, Adana, Türkiye; 4 Department of Chest Diseases, Çukurova University Faculty of Medicine, Adana, Türkiye; 5 Başakşehir Çam Sakura Hospital, Emergency Medicine Clinic, İstanbul, Türkiye

**Keywords:** Acute kidney injury, creatinine, NSAIDs, renal replacement therapy

## Abstract

**Background:**

Acute kidney injury (AKI) is a common condition in emergency departments (EDs), often associated with significant mortality and morbidity. This study aimed to identify predictors of renal replacement therapy (RRT) requirement in patients presenting with AKI who did not meet absolute indications for RRT at ED evaluation but subsequently required RRT during follow-up.

**Methods:**

A total of 266 patients with AKI who were assessed in the ED were enrolled in this prospective observational study and subsequently monitored in the Internal Medicine Intensive Care Unit (ICU) between October 2022 and September 2023. Patients were allocated into 2 analytically defined categories: those not requiring renal replacement therapy [RRT (−)] and those who required RRT [RRT (+)]. Laboratory and clinical data were recorded prospectively.

**Results:**

During follow-up, 30.8% of the patients (n = 82) required RRT due to complications. The overall mortality rate of the study cohort was 32.3%. Regression analysis identified nonsteroidal anti-inflammatory drug (NSAID) use (OR: 7.944, 95% CI: 1.583-39.871, *P* = .012) and elevated creatinine levels (OR: 1.321, 95% CI: 1.017-1.715, *P* = .037) as independent predictors of RRT requirement. Receiver operating characteristic analysis revealed that the area under the curve for creatinine levels was 0.818 (95% CI: 0.761-0.874, *P* < .001). A creatinine cut-off value of 3.15 mg/dL was determined, with a sensitivity of 79.3% and a specificity of 70.1%.

**Conclusion:**

NSAID use and elevated serum creatinine at ED presentation are independent predictors of early RRT requirement in AKI patients. These factors may assist clinicians in identifying high-risk patients who may benefit from closer monitoring or earlier intervention.

**Main Points**

- Acute kidney injury (AKI) is a serious and common problem in emergency and critical care, associated with high mortality and morbidity.
- The timing of initiating renal replacement therapy (RRT) in AKI is controversial: early initiation may prevent life-threatening complications but risks unnecessary procedures, while delayed initiation risks progression to severe complications.
- This study shows that nonsteroidal anti-inflammatory drug use and elevated creatinine at emergency department presentation are strong predictors of early RRT need.
- A creatinine cut-off of 3.15 mg/dL provides practical guidance for identifying high-risk AKI patients who require closer monitoring and timely intervention.

## Introduction

Acute kidney injury (AKI) is a complex clinical condition characterized by the sudden loss of renal function.^1^^,^^2^ It can develop through prerenal, intrarenal, and postrenal mechanisms, leading to the inability to excrete urea, creatinine, and other uremic toxins, reduced urine output, and disrupted electrolyte balance.^3^ AKI is common in severely ill patients and increases mortality and morbidity.^1^^,^^4^ Despite advances in treatment and the use of renal replacement therapy (RRT), AKI is associated with a poor prognosis in severely ill patients.^5^ Managing this condition includes conservative interventions such as circulatory resuscitation while avoiding excessive volume overload and nephrotoxic agents.^1^ If these interventions are ineffective, patients can be treated with RRT.^1^^,^^4^ The timing of initiating RRT in the management of severe AKI is uncertain.^2^^,^^3^^,^^4^^,^^6^ Serious electrolyte disturbances—including hyperkalemia, profound metabolic acidosis, or uremic encephalopathy—are generally considered indications for the prompt initiation of RRT.^3^^,^^6^^,^^7^ These are life-threatening complications of AKI. Initiating RRT before these life-threatening complications occur may benefit patients with AKI by correcting electrolyte imbalances, maintaining fluid balance and acid-base homeostasis, and reducing exposure to the metabolic risks of untreated AKI.^2^^,6^ Nonetheless, the most suitable point at which to start RRT remains ambiguous when such problems do not occur.^8^^-^^10^ Additionally, early initiation of RRT in these patients may lead to the overlooking of the group of patients whose renal functions would recover without such treatment. Therefore, determining whether earlier initiation improves outcomes is crucial for patient care, considering the risks associated with RRT.^2^^,^^11^^,12^ Randomized trials evaluating early versus postponed initiation of RRT in severely ill patients with AKI have yielded mixed and often conflicting results. These studies have primarily focused on the timing of complications requiring RRT after the diagnosis of AKI.^2^^,3^ Establishing evidence-based criteria for the appropriate and safe initiation of RRT can help rationalize the use of this costly treatment.^3^

This study aimed to determine clinical features that predict the need for renal replacement therapy during follow-up among patients presenting to the emergency department (ED) with acute kidney injury who did not initially fulfill standard absolute indications for RRT.

## Material and Methods

The research was carried out as a prospective observational case series within the ED of a tertiary care center. Patients diagnosed with AKI in the emergency department were subsequently transferred to the Internal Medicine Intensive Care Unit for ongoing evaluation and treatment. The primary objective of the study was to identify clinical factors associated with the subsequent need for renal replacement therapy in patients presenting with AKI who did not demonstrate absolute indications for RRT at initial emergency department assessment. In the present study, the term “early renal replacement therapy” is used to describe patients who did not meet absolute indications for RRT at the time of emergency department presentation but subsequently required RRT during follow-up due to the progression of AKI or the development of complications, rather than to indicate a time-based initiation strategy. Patients requiring immediate RRT at admission were excluded to ensure that the analysis focused on predictors identifiable before the development of established absolute indications for RRT.

The study commenced following approval from the institutional ethics committee (Meeting Date: 08.09.2022; Meeting Number: 111; Decision No: 2116) and was conducted in accordance with the principles of the Helsinki Declaration and established standards of good clinical practice. Written informed consent was obtained from the patients who agreed to take part in the study.

### Patient Selection

**Figure 1.**
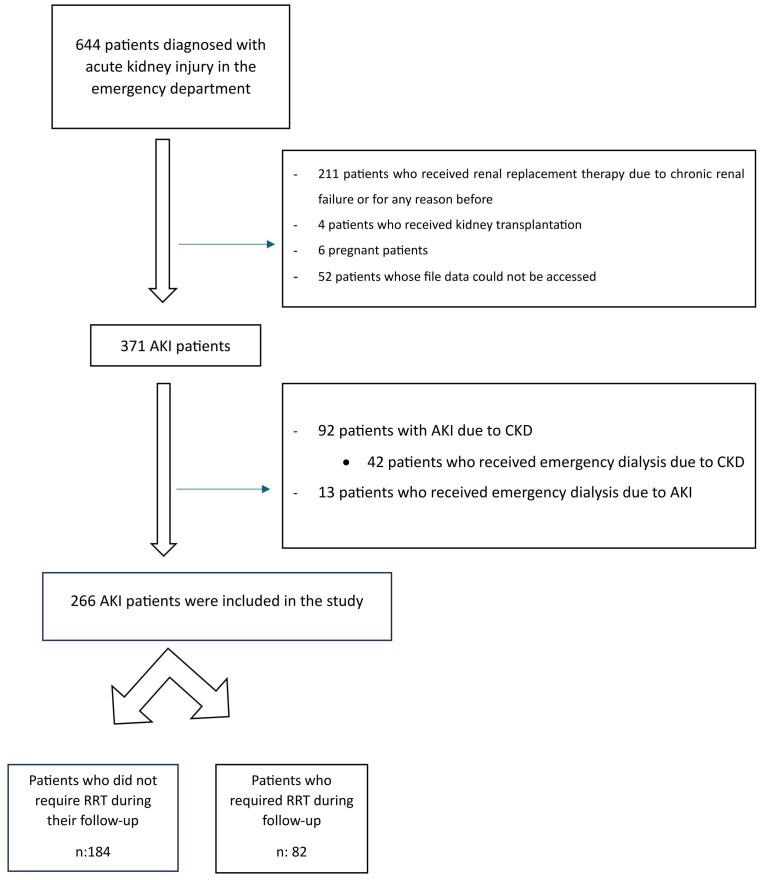
Flow chart of patients included in the study.

Participants aged 18 years or older who were diagnosed with AKI at ED admission between October 1, 2022 and September 30, 2023, were considered for inclusion. Only patients with complete clinical data were included. The sample size was justified based on the prevalence of AKI in the ED and the need for a representative sample. Exclusion criteria were:

- Age under 18 years
- Immediate requirement for RRT at the time of ED assessment
- Pre-existing chronic kidney disease or end-stage renal failure
- History of renal replacement therapy for any indication
- Previous kidney transplantation
- Pregnancy
- Incomplete medical records (Figure 1)

### Study Design

AKI at ED presentation was identified according to the KDIGO criteria.^13^ These criteria were met when one of the following conditions was present:

- A rise in serum creatinine of ≥0.3 mg/dL within a 48-hour interval
- An increase of ≥1.5 times the known or estimated baseline creatinine level within the preceding 7 days
- A urine output persistently below 0.5 mL/kg/hour for at least 6 hours

According to the KDIGO classification,^13^ AKI staging was defined as follows:

- Stage 1 was characterized by an increase in serum creatinine of 1.5-1.9 times baseline or an absolute increase of ≥0.3 mg/dL, or a urine output <0.5 mL/kg/h for 6-12 hours.
- Stage 2 was defined as an increase in serum creatinine of 2.0-2.9 times baseline or a urine output <0.5 mL/kg/h for ≥12 hours.
- Stage 3 was defined as an increase in serum creatinine to ≥3.0 times baseline, an absolute serum creatinine level ≥4.0 mg/dL, initiation of renal replacement therapy, or a urine output <0.3 mL/kg/h for ≥24 hours or anuria for ≥12 hours.

The clinical and demographic characteristics, presenting complaints, medications used, admission diagnoses, laboratory parameters, RRT needs, and outcomes of the included AKI patients were recorded on a standard data form. The need for RRT was determined by a nephrology specialist. Additionally, progression to chronic kidney failure (CKD) requiring long-term dialysis during the 3-month follow-up period was recorded.

The main goal of the study was to identify the clinical determinants associated with the need for RRT during the follow-up of patients presenting to the ED with a diagnosis of AKI. For this purpose, the cohort was divided into 2 groups according to subsequent RRT use: individuals who did not require RRT [RRT (−)] and those who eventually required it [RRT (+)].

### Statistical Analysis

Continuous variables were evaluated using descriptive statistics, and categorical variables were summarized as proportions and frequencies. The distribution of quantitative data was assessed through the Kolmogorov–Smirnov test together with visual inspection methods. Comparisons between normally distributed variables were performed using the Student’s *t*-test, while non-normally distributed variables were analyzed with the Mann–Whitney *U* test. Categorical variables were compared with the Chi-square test. To identify independent predictors of renal replacement therapy, a binary logistic regression model was constructed using demographic characteristics, presenting clinical information, medication history, and laboratory findings obtained at emergency department admission. Significant variables from the regression model were further evaluated using receiver operating characteristic (ROC) analysis to quantify their diagnostic performance. Optimal cut-off values were derived using the Youden index, prioritizing the point with the highest combined specificity and sensitivity. Diagnostic accuracy metrics, including sensitivity, specificity, and corresponding 95% confidence intervals, were tabulated. All analyses were performed using SPSS version 25 (SPSS Inc., Chicago, IL, USA). A *P*-value of <.05 was considered statistically significant.

## Results

From October 1, 2022, to September 30, 2023, a total of 644 adults (≥18 years) requiring intensive care presented to the emergency department and were identified as having AKI. After applying the predefined exclusion criteria, 266 patients met the eligibility requirements and were included in the final analysis (Figure 1).

According to the KDIGO classification, the stages of AKI in the patients (N = 266) were as follows: 43.6% (n = 116) were Stage 1; 16.2% (n = 43) were Stage 2; 40.2% (n = 107) were Stage 3. During follow-up, 30.8% of patients (n = 82) required RRT. The mortality rate among the patients was 32.3% (n = 86). Among the surviving patients (n = 180), at the 3-month follow-up: 65% (n = 117) returned to normal kidney function; 26.1% (n = 47) developed chronic kidney disease (CKD); 7.8% (n = 14) were included in a routine dialysis program due to chronic kidney failure (CKF).

50.8% (n = 135) of the patients were female, and the average age was 71 years (IQR: 59.8-79). Neither age nor sex was significantly associated with the requirement for renal replacement therapy (*P* = .637 and *P* = .487, respectively). Median central venous pressure (CVP) [3 cm H_2_O (IQR: 0-10)] was significantly higher in patients who required RRT (*P* = .008). The most common comorbidities among the patients were: hypertension (HT) at 58.6%, diabetes mellitus (DM) at 38.7%, coronary artery disease (CAD) at 33.8% (Table 1).

The most common presenting complaints were: oral intake disorders at 31.6%, shortness of breath at 27.4%, nausea-vomiting at 22.6%. The most common admission diagnoses were: sepsis at 20.7%, pneumonia at 17.3%, urinary tract infection at 15%. The most commonly used medications were: antidiabetics at 28.2%, hydrochlorothiazide at 28.2%, ARBs at 27.8%. RRT requirement was significantly more frequent among patients using NSAIDs (*P* = .002) and metformin (*P* = .022) (Table 1).

Patients who required RRT exhibited significantly *lower* values of hemoglobin (*P* = .007), hematocrit (*P* = .021), GFR (*P* < .001), pH (*P* < .001), HCO_3_ (*P* < .001), base excess (*P* < .001), and lactate (*P* = .031). In contrast, several laboratory markers were significantly *elevated* in this group, including creatinine (*P* < .001), urea (*P* < .001), potassium (*P* < .001), BNP (*P* < .001), magnesium (*P* = .013), phosphorus (*P* = .023), and uric acid (*P* < .001). Additional laboratory parameters are presented in Table 2.

Binary logistic regression analysis was performed to predict the need for RRT. According to the regression model, both NSAID exposure (OR: 7.944, 95% CI: 1.583-39.871; *P* = .012) and increased serum creatinine (OR: 1.321, 95% CI: 1.017-1.715; *P* = .037) emerged as independent determinants of renal replacement therapy requirement (Table 3).

**Figure 2.**
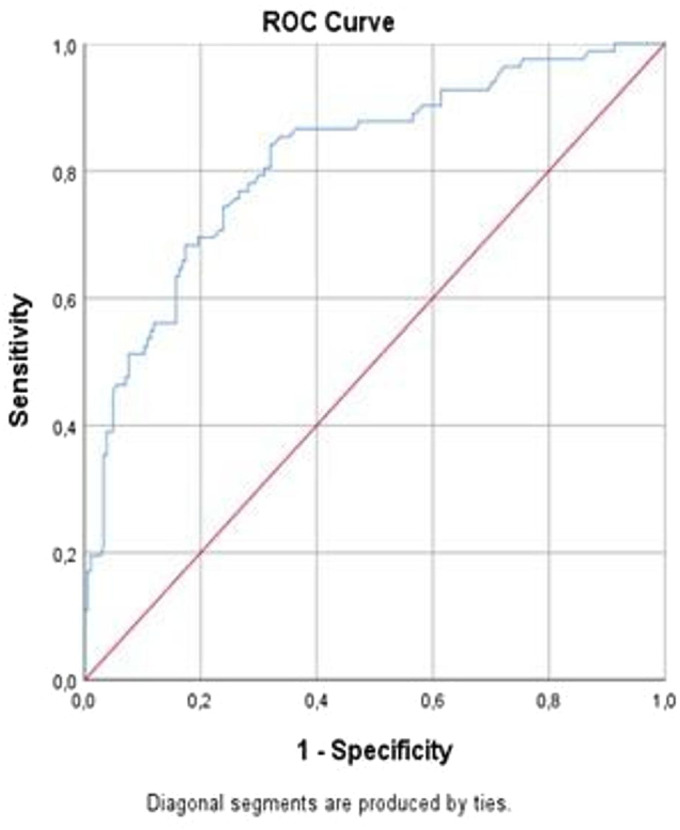
ROC curve of the predictive power of creatinine for the need for RRT.

ROC curve analysis was performed to assess the discriminative ability of the significant logistic regression variables. Figure 2 illustrates the ROC curves. Creatinine level demonstrated the strongest predictive performance, with an area under the curve of 0.818 (95% CI: 0.761-0.874, *P* < .001). A creatinine cut-off value of 3.15 mg/dL yielded a specificity of 70.1% and a sensitivity of 79.3%.

## Discussion

In this study, the 2 most important independent factors predicting the need for renal replacement therapy in patients presenting to the ED with AKI were the use of NSAIDs and elevated serum creatinine levels. Specifically, a creatinine level above 3.15 mg/dL showed high sensitivity (79.3%) and specificity (70.1%) in determining the need for RRT. The data emphasize that these 2 parameters should be more thoroughly incorporated into the ED evaluation process.

AKI remains a complex and critical condition with high mortality despite advances in treatment, including the use of RRT.^5^ Even with RRT, high mortality can still be independently associated with critical patient groups.^14^ This complexity often leaves clinicians in a dilemma about when to initiate RRT in a patient with AKI.^15^ There is no universal definition for early or late initiation of RRT, which complicates the decision-making process for clinicians and emphasizes the importance of individualized treatment approaches.

Early initiation of RRT can help control acid-base and electrolyte imbalances, manage hypervolemia early, eliminate uremic toxins, reduce systemic and renal inflammatory responses, and promote early organ function recovery.^4^ Prospective and retrospective studies based on this approach have shown that early renal function recovery in patients can reduce hospital stay and mortality.^16^^-^^18^ Nevertheless, it should be emphasized that unnecessary early RRT can increase the risk of hypotension, catheter-related infections, and other complications associated with RRT. Late initiation of RRT, on the other hand, is typically started in situations with absolute indications (such as uremic pericarditis or encephalopathy, treatment-resistant hypervolemia, treatment-resistant electrolyte imbalance, or treatment-resistant metabolic acidosis) or in advanced stages of AKI with high serum urea and creatinine levels.^19^ Late RRT can help control hemodynamic and pulmonary parameters, reduce catheter-related complications, prevent complications associated with RRT itself, and allow for renal recovery, potentially eliminating the need for RRT.^4^ However, delays in initiating RRT can negatively impact the patient's overall prognosis.

Key factors influencing the timing of RRT initiation include evidence of disease severity and organ function impairment, as well as healthcare providers' subjective perceptions of the benefit-risk relationship. This variability can lead to different decisions regarding the timing of RRT initiation in different clinical settings and approaches. The studies evaluating the timing and outcomes of RRT initiation have produced conflicting results due to uncontrolled variations in clinical approaches, such as very low survival rates in severely ill patients, high RRT use in patients with a high likelihood of renal recovery, differences in the quality of care in different critical care environments, and differences in the clinical characteristics and disease severity of included patients.^20^ Physicians face the challenging balance of deciding between early and late initiation of RRT in AKI patients. Early initiation of RRT may expose the patient to unnecessary treatment, potential complications, and high costs, while also potentially preventing the natural recovery of renal function. Conversely, late initiation of RRT may increase the risk of developing serious, life-threatening complications. Therefore, the timing of RRT is a critical decision that requires careful evaluation for each patient.

Numerous studies have been conducted on early and late RRT. The ELAIN study showed that early RRT in post-cardiac surgery patients with stage 2 AKI reduced mortality and hospital stay compared to those who developed stage 3 AKI and received late RRT.^12^ In contrast, the concurrent AKIKI study found that early RRT increased the rates of hypophosphatemia and catheter-related infections in about half of the patients.^11^ Subsequent studies, such as IDEAL-ICU,^8^ STARRT-AKI,^2^ and AKIKI 2,^21^ reported similar findings, showing that renal function recovered spontaneously in about half of the patients with severe AKI who received late RRT.

Numerous biomarkers have been investigated to clarify their usefulness in guiding the optimal initiation of renal replacement therapy. A large meta-analysis involving 15,000 patients and 13 biomarkers reported that several indicators—such as neutrophil gelatinase-associated lipocalin, cystatin C, serum creatinine, tissue inhibitor of metalloproteinases-2, urinary interleukin-18, and insulin-like growth factor binding protein-7—may aid in predicting when RRT should be started.^22^ Despite these promising findings, no universally accepted standard has yet been defined.^3^ Even if such benchmarks are established through future research, widespread adoption in clinical practice will likely require considerable time.

This study focused on patients admitting to the ED with a diagnosis of AKI. Patients who required RRT at the time of presentation were excluded from the study. Patients who did not require RRT during their standard treatment were compared with those who developed complications requiring RRT during their follow-up. The data demonstrated that NSAID use and elevated serum creatinine levels were independent predictors of subsequent renal replacement therapy requirement in patients with acute kidney injury. NSAID use increased the need for RRT by 7.9 times, while each unit increase in creatinine increased the need by 1.3 times. Using a cut-off value of 3.15 mg/dL for creatinine, the sensitivity was 79.3% and the specificity was 70.1%. Serum creatinine is still used as a biomarker in AKI classifications due to its ease of measurement and low cost. AKI severity is determined by the increase in serum creatinine and the corresponding decrease in urine output. However, it is important to remember that creatinine reflects renal function rather than actual kidney damage. Due to the proximal tubular secretion of creatinine, glomerular filtration rate is considered an indicator of kidney damage when measured by creatinine clearance.^23^ Factors such as muscle mass, age, gender, race, medications, diet, nutritional status, fluid loading, and sepsis can affect creatinine levels. Nevertheless, serum creatinine remains an important biomarker for AKI.

NSAIDs are among the most frequently used medications worldwide, and their association with acute kidney injury has been extensively examined. Evidence from meta-analyses and observational studies indicates that conventional NSAIDs are associated with an increased risk of AKI.^24^^-^^26^ These agents exert their effects by inhibiting the cyclooxygenase enzyme, which catalyzes the conversion of phospholipids into prostaglandins. Suppression of prostaglandin synthesis can lead to several renal consequences, including renal vasoconstriction. Reduced prostaglandin activity also interferes with salt and water handling, thereby promoting edema and elevated blood pressure. Additional NSAID-related complications include hyponatremia, hyperkalemia, acute interstitial nephritis, and nephrotic syndrome. Given these risks, minimizing NSAID exposure is particularly important in individuals at increased risk of renal impairment.^27^ Beyond their association with AKI development, nonsteroidal anti-inflammatory drugs may contribute to the progression of AKI by disrupting renal autoregulation through inhibition of prostaglandin-mediated afferent arteriolar vasodilation, particularly in patients with reduced effective circulating volume. Under conditions of renal hypoperfusion, prostaglandins play a critical compensatory role in maintaining glomerular filtration. NSAID-induced suppression of this mechanism may accelerate the decline in glomerular filtration rate, thereby increasing AKI severity and the likelihood of subsequent renal replacement therapy requirement. This effect may be especially pronounced in emergency department patients, who frequently present with dehydration, sepsis, or hemodynamic instability, where NSAID exposure can further exacerbate renal hypoperfusion and limit spontaneous renal recovery.^24^

### Limitations

This study has several strengths, including its prospective design and the use of ROC analysis to assess predictive performance. However, certain limitations should be acknowledged. Because the research was carried out at a single institution and relied on a relatively modest number of participants, the applicability of the findings to broader patient groups or different clinical environments may be restricted. This constraint could also reduce the statistical power to identify uncommon predictors of RRT requirement. Variability in clinician decision-making and institutional policies regarding the initiation of RRT may have introduced additional confounding factors. Moreover, differences in the types, dosages, and duration of NSAID exposure—factors that may influence AKI progression—were not assessed in this study.

## Conclusion

A history of NSAID exposure and elevated serum creatinine levels are strong predictors of subsequent renal replacement therapy requirement among patients presenting to the emergency department with acute kidney injury. Recognizing these indicators promptly may allow clinicians to identify individuals at heightened risk and improve subsequent therapeutic decisions. The data indicate that incorporating these parameters into routine clinical judgment has the potential to optimize patient management strategies. In addition, the findings may inform the creation of updated protocols applicable to both emergency departments and intensive care settings. Moving forward, large-scale, prospective multicenter investigations—particularly those integrating biomarker assessments—are needed to strengthen external validity and further refine clinical guidelines.

## Figures and Tables

**Table 1.** Comparison of Demographic and Clinical Data of Patients

	Total N = 266	RRT (−) n = 184 (63.1%)	RRT (+) n = 82 (36.9%)	*P*
Sex				
Female	135 (50.8%)	96 (52.2%)	39 (47.6%)	.487
Male	131 (49.2%)	88 (47.8%)	43 (52.4%)	
Age (years)	71 (59.8-79)	71 (60.5-79)	70 (57-79)	.637
Vital sign				
Temperature (°C)	36.5 (36.2-36.8)	36.5 (36.2-36.8)	36.5 (36.2-36.8)	.454
Respiratory rate (min)	16 (16-19)	16 (16-19)	16 (15.5-18)	.159
Pulse (min)	89 (77-102.8)	89 (76-103)	88 (78.5-101.5)	.480
Mean arterial pressure (mmHg)	86.7 (73.3-96.7)	83.3 (73.3-96.7)	86.7 (75-100)	.159
Oxygen saturation (%)	96 (92-98)	96 (92-98)	96 (93.5-98)	.134
Glasgow Coma Scale	15 (15-15)	15 (15-15)	15 (14-15)	.472
Central venous pressure	2 (0-6)	2 (0-5)	3 (0-10)	.008^a^
Comorbidities				
Hypertension	156 (58.6%)	104 (56.5%)	52 (63.4%)	.292
Diabetes mellitus	103 (38.7%)	72 (39.1%)	31 (37.8%)	.838
Coronary artery disease	90 (33.8%)	63 (34.2%)	27 (32.9%)	.835
Cancer	52 (19.5%)	35 (19%)	17 (20.7%)	.745
Chronic heart failure	47 (17.7%)	36 (19.6%)	11 (13.4%)	.225
Cerebrovascular disease	33 (12.4%)	26 (14.1%)	7 (8.5%)	.201
Chronic obstructive pulmonary disease	28 (10.5%)	22 (12%)	6 (7.3%)	.255
Dementia	21 (7.9%)	18 (9.8%)	3 (3.7%)	.087
Chronic liver failure	5 (1.9%)	5 (2.7%)	0	.132
Admission complaint				
Oral intake disorder	84 (31.6%)	56 (30.4%)	28 (34.1%)	.548
Shortness of breath	73 (27.4%)	46 (25%)	27 (32.9%)	.181
Nausea-vomiting	60 (22.6%)	42 (22.8%)	18 (22%)	.875
Weakness	42 (15.8%)	26 (14.1%)	16 (19.5%)	.266
Abdominal pain	41 (15.4%)	30 (16.3%)	11 (13.4%)	.547
Inability to urinate	32 (12%)	21 (11.4%)	11 (13.4%)	.643
Changes in consciousness	29 (10.9%)	19 (10.3%)	10 (12.2%)	.652
Gastroenteritis	18 (6.8%)	13 (7.1%)	5 (6.1%)	.772
Fever	16 (6%)	13 (7.1%)	3 (3.7%)	.281
Dizziness	3 (1.1%)	2 (1.1%)	1 (1.2%)	.925
Syncope	2 (0.8%)	2 (1.1%)	0	.343
Diagnoses				
Sepsis	55 (20.7%)	32 (17.4%)	23 (28%)	.047^a^
Pneumonia	46 (17.3%)	34 (18.5%)	12 (14.6%)	.444
Urinary tract infection	40 (15%)	27 (14.7%)	13 (15.9%)	.804
Acute heart failure	33 (12.4%)	21 (11.4%)	12 (14.6%)	.462
Pyelonephritis	20 (7.5%)	11 (6%)	9 (11%)	.153
Cellulite	16 (6%)	12 (6.5%)	4 (4.9%)	.603
Ileus	18 (4.9%)	13 (5.6%)	5 (3.6%)	.410
Acute cerebrovascular disease	10 (3.8%)	7 (3.8%)	3 (3.7%)	.954
Acute coronary syndrome	6 (2.3%)	3 (1.6%)	3 (3.7%)	.304
Acute liver failure	5 (1.9%)	5 (2.7%)	0	.132
Cholangitis	5 (1.9%)	3 (1.6%)	2 (2.4%)	.654
Contrast induced acute kidney injury	4 (1.5%)	1 (0.5%)	3 (3.7%)	.054
Cholecystitis	3 (1.1%)	3 (1.6%)	0	.245
Pancreatitis	2 (0.8%)	1 (0.5%)	1 (1.2%)	.556
Drugs used				
Antidiabetic drugs	75 (28.2%)	57 (31%)	18 (22%)	.131
Hydrochlorothiazide	75 (28.2%)	51 (27.7%)	24 (29.3%)	.795
Angiotensin 2 receptor blockers	74 (27.8%)	52 (28.3%)	22 (26.8%)	.810
Furosemide	64 (24.1%)	47 (25.5%)	17 (20.7%)	.397
Angiotensin-converting enzyme inhibitors	64 (24.1%)	42 (22.8%)	22 (26.8%)	.481
Calcium channel blockers	47 (17.7%)	34 (18.5%)	13 (15.9%)	.604
Metformin	36 (13.5%)	19 (10.3%)	17 (20.7%)	.022^a^
Aldactone	32 (12%)	25 (13.6%)	7 (8.5%)	.242
Non-steroidal anti-inflammatory drugs	13 (4.9%)	4 (2.2%)	9 (11%)	.002^a^
Antidepressant	5 (1.9%)	3 (1.6%)	2 (2.4%)	.654

RRT (−), patients who do not need renal replacement therapy requirement; RRT (+), patients who develop renal replacement therapy requirement.

^a^Statistical significance.

**Table 2.** Comparison of Laboratory Parameters of Patients

	Total N = 266	RRT (−) n = 184 (63.1%)	RRT (+) n = 82 (36.9%)	*p*
Leukocyte (10³/µL)	12 (8.4-17.1)	11.7 (8.2-16.8)	12.6 (8.7-18.7)	.378
Hemoglobin (g/dL)	11 (9.4-12.7)	11.2 (9.8-12.9)	10.4 (8.7-12)	.007^a^
Platelet (10³/µL)	247 (173.8-24.5)	252 (174-323.5)	236.5 (166.5-332.3)	.783
Hematocrit^b^ (10³/µL)	33.5±7.2	34.2±6.8		.021^a^
Glucose (mg/dl)	123 (94.8-182.5)	125.5 (96-182)	32±7.9	.882
Sodium (mmol/L)	135 (131-139)	135 (131-138.8)	117.5 (92-191.3)	.556
Chloride (mmol/L)	101 (95-106)	100 (95-105)	136 (131-140)	.458
Potassium (mmol/L)	4.8 (4.1-5.7)	4.6 (3.9-5.3)	102.5 (95.8-108)	<.001^a^
Calcium(mg/dl)	8.6 (8-9)	8.6 (8.1-9)	5.7 (4.7-6.4)	.132
Aspartate aminotransferase (U/L)	29 (18.8-61.3)	18 (11-38.8)	8.4 (7.9-9.1)	.190
Alanine aminotransferase (U/L)	18 (11-41)	29 (19-54.8)	18 (11-45.5)	.547
Urea (mg/dl)	127.5 (86.5-177.5)	110.5 (75-	29.5 (18-82.3)	<.001^a^
Creatinine (mg/dl)	2.9 (2.03-4.7)	151.8)	183(121.5-267.3)	<.001^a^
Albumin (g/L)	31 (26-36)	2.3 (1.9-3.3)	5.4 (3.3-8.2)	.354
C-reactive protein (mg/L)	109 (25-198.5)	31(26.3-35)	31.5 (26-36)	.901
B-Natriuretic Peptide (µg/L)	3456 (978.5-10665)	107.5 (24-199.5)	109 (28.3-196)	<.001^a^
Magnesium (mg/dL)	2 (1.7-2.3)	2607 (792-	5058 (2351.5-	.013^a^
Phosphorus	4.8 (3.6-6.3)	7540)	23608.5)	.023^a^
Uric Acid (mg/dL)	8.9 (7.2-11.5)	2 (1.7-2.2)	2.1 (1.8-2.5)	<.001^a^
Glomerular filtration rate	19 (11-27.3)	4.4 (3.4-5.4)	6.2 (5-7.8)	<.001^a^
Plasma osmolarity	279 (271-289.3)	8.5 (6.9-10.6)	10.4 (7.9-12.8)	.581
Prothrombin Time (min)	14.4 (12.9-16.7)	22 (15-31)	9 (5-15)	.431
Activated partial thromboplastin time (minutes)	27.9 (24.3-33.9)	279 (271.3-	280 (271-291)	.537
International normalized rate	1.18 (1.06-1.39)	1.16 (1.03-1.33)	1.23 (1.11-1.40)	.954
Ph	7.30 (7.24-7.37)	7.33 (7.27-7.38)	7.23 (7.17-7.28)	<.001^a^
Bicarbonate	18.9 (15.3-22.7)	20.9 (17.5-24)	15 (12.5-17.4)	<.001^a^
Base excess^ b^	-6.6±8.4	-4.02±7.2	-12.5±8.1	<.001^a^
Lactate (mmol/L)	18 (12-31.3)	18 (12-30.8)	16 (10.8-46)	0.031^a^

RRT (−), patients who do not need renal replacement therapy requirement; RRT (+), patients who develop renal replacement therapy requirement.

^a^Statistical significance.

^b^Mean ± standard deviation.

**Table 3.** Binary Logistic Regression Analysis Performed to Predict the Need for RRT in Patients

	**OR**	**95% CI** **Lower Limit-Upper Limit**	***P***
NSAIDs use Creatinine Metformin use Potassium Magnesium CVP Uric acid Urea Hematocrit	7.944 1.321 1.414 1.173 1.156 1.056 1.042 1.006 1.003	1.583-39.871 1.017-1.715 0.512-3.901 0.810-1.697 0.775-1.724 0.997-1.117 0.926-1.173 0.999-1.013 0.838-1.200	.012^a^ .037^a^ .504 .398 .476 .061 .495 .069 .977

For RRT requirement, the following variables were entered: MAP, CVP, CKD (1), Dyspnea (1), NSAID Use (1), AHF (1), HB, HCT, K, Urea, Cre, BNP, Uric acid, GFR, T4, PH, HCO3, BE, CKMB.

For mortality, the following variables were entered: Age, respiratory rate, pulse, MAP, GCS, SaO2, dyspnea (1), oral intake disorder (1), change in consciousness (1), sepsis (1), pneumonia (1), urinary tract infection (1), cerebrovascular disease (1), liver failure (1), Wbc, Hct, Na, Ast, Urea, Cre, Total protein, Albumin, BNP, Crp, phosphorus, Mg, CKMB, PTZ, Ph, BE, Lactate.

OR, odds ratio (relative probability ratio); OR calculated according to logistic regression analysis is for every 1 unit increase.

^a^Statistical significance.

CVP, central venous pressure; NSAID, non-steroidal anti-inflammatory drugs.

## Data Availability

The data that support the findings of this study are available on request from the corresponding author.
